# Evolution-revolution-devolution: a short history of the provision of knowledge-based information services to Manitoba’s health professionals

**DOI:** 10.29173/jchla29510

**Published:** 2021-08-01

**Authors:** Ada Ducas, Tania Gottschalk, Analyn Cohen-Baker

**Affiliations:** 1 Librarian Emerita. Former Head, Head, Health Sciences Libraries. University of Manitoba, Winnipeg MB; 2 Interim University Librarian. Thompson Rivers University, Kamloops, BC; 3 Retired Librarian. Former Head, Seven Oaks Hospital Library, Winnipeg, MB

## Abstract

Since 1993, the University of Manitoba (UM), Winnipeg area hospitals, the Winnipeg Regional Health Authority (WRHA), and the Manitoba Health Department have engaged in a series of agreements that have changed access to knowledge-based information for health professionals. These agreements gradually transferred the management and delivery of library service from hospital libraries to the UM Libraries. This paper describes the historical evolution in health information access in Winnipeg, subsequent revolutionary changes that resulted in the Health Sciences Libraries Service Model, and the devolution of the model following serious challenges. Its rebirth as the WRHA Virtual Library is discussed with factors that may impact the new service model.

## Introduction

Library programs and services are products of their environments. Political, educational, structural, and technological factors at play within individual provinces reflect the relationships that governments develop with health sciences libraries and their clients, typically health professional schools and health care practitioners. [1,2]. Manitoba has one major city, Winnipeg; one MD/Ph.D. granting institution, the University of Manitoba (UM); one faculty of health sciences, and one significant health sciences library, the UM Neil John Maclean Health Sciences Library (NJMHSL). These particulars shaped a unique “made in Manitoba” solution for the delivery of library services to the health care community in the province. This paper describes the events that led to the evolution of health information access in Winnipeg, subsequent revolutionary changes in these services, and their eventual devolution due to an array of significant challenges.

## Part 1: Evolution

### The Early Years of Starts and Stops

Manitoba has a history of delivering centrally supported, knowledge-based information services to health professionals. The UM’s original Medical Library was established in 1920, when the College of Physicians and Surgeons of Manitoba (CPSM) gifted their library to the University with a legal agreement stating “The College members would have at all reasonable hours, access to the said library and all addition hereto” and that “the privilege which is at present enjoyed by out-of-town members of the medical profession of receiving books by post….Shall be continued and shall apply to all additions to the Library” [3]. In the 1970s and 1980s, the Head of the Medical Library, Audrey Kerr, spearheaded programs to coordinate delivery of information to medical professionals. In 1975, CPSM granted funds to the Medical Library for library services to rural physicians. In 1976, the *Medical Library Extension Service* was inaugurated with an Extension Librarian. Although the program was heavily used and valued by CPSM members, funding ceased in 1993. Mandate changes required CPSM funds to be used only “to expand programs for which it was directly accountable in the areas of licensure, standards of medical practice, and discipline” [4].

In 1980, the Manitoba Health Libraries Association (MHLA) established a Task Force on Shared Services to review the provision of health information and to investigate ways in which it could be improved and expanded through cooperative programs and services [5]. The Task Force recommended the “appointment of an area coordinator to maintain existing shared services that have been developed by MHLA; and to provide technical service back-up to the member libraries and reference services including Medline” [5]. In 1983, in response to these recommendations and with one-time funding from the Winnipeg Foundation, the *Winnipeg Health Information Network* (WHINET) pilot project was launched. The 18-month WHINET project was successful but as permanent funding could not be secured, it was abandoned. [6]

In September 1988, the Manitoba Association of Registered Nurses (MARN) closed its library because the library was under-used and MARN could not budget for materials, technology, or staffing. MARN members were told to use hospital libraries or the MHLA network of libraries. Nurses in Winnipeg and Brandon hospitals had access to some resources but nurses in rural areas were left without services. Not only were nurses underserved but library services for rural hospital administrators and allied health personnel were nonexistent. In 1989, Ada Ducas, then the Director of Educational Resources at the Health Sciences Centre (HSC), laid the groundwork to establish the Manitoba Health Information Network (MHINET) to meet the information needs of nurses and health administrators. Presentations were made to the Manitoba Health Organizations Inc. (MHO) and MARN. MHINET was approved as a two-year demonstration project by both organizations and the HSC [7]. The role of MHINET was to meet the information needs of Manitoba nursing professionals, hospitals administrators, and allied health professionals. MHINET services delivered through the HSC Library Services department included a toll-free number; access to book and journal collections in the UM Medical and HSC libraries; reference and consultation services; computerized literature searches; and a current awareness service.

The MHINET demonstration project was very successful and MARN agreed to continue funding the service for nurse members and it operated from 1989 to 2003. In 2001, the passage of The Registered Nurses Act, changed MARN’s mandate to a regulatory body. With the establishment of the College of Registered Nurses of Manitoba (CRNM) funding for MHINET was discontinued despite a report outlining the benefits and value of the MHINET service [8]. By this time, the MHO had been replaced by the Manitoba Health Department, which was also not interested in funding the service. In 2003, MHINET services ceased.

These promising starts and stops to the provision of library services demonstrated that sustained and ongoing funding was critical in order to improve information access for health professionals in Winnipeg and across the province.

### Factors Leading to Change

#### a. *Technological*

In the 1980s and 1990s, technological, educational, and political factors triggered new thinking around information access for health professionals. Winnipeg hospital libraries ranged from a state-of-the-art facility located in a research centre to basement rooms with inadequate staffing and collections. Limited coordination of library services was a concern noted in a 1998 MHLA position paper: “Better coordination of health sciences information resources has also been hampered by the diversity of mandate, governance and funding amongst libraries” [6]. Despite these challenges, MHLA developed a number of initiatives such as the publication of the *Selected Books and Journals for Manitoba Health Care Facilities* in 1979-80 and, as early as 1978-79, the *Serials Holdings of Member Libraries* [9]. This computerized union list included locations of 800+ journal titles held by sixteen hospital and health libraries in Winnipeg and Brandon. Additionally, staff of the HSC Library Services maintained a card catalogue of books held in Winnipeg’s hospital libraries.

However, technology was rapidly changing with the introduction of service delivery methods such as DOCLINE and the Internet. In Winnipeg, only the three hospital libraries staffed with professional librarians, were integrating technology into their services and had adequate collection budgets: the HSC, St. Boniface General Hospital (SBGH) and Deer Lodge Centre. Smaller hospital libraries (Victoria, Seven Oaks, Grace, Concordia, Misericordia, and Riverview) were disadvantaged as they had part-time library technicians, inadequate collections budgets and limited access to new technologies. In 1995-96, an MHLA ad-hoc committee conducted a survey to determine technical and financial resources needed to implement DOCLINE in Winnipeg hospital libraries and found that several libraries had no computer equipment or Internet access [6]. Outside of Winnipeg, the state of the hospital libraries was worse.

#### b. *Educational*

New education needs were also driving change. In 1988, MARN supported a resolution “That by the year 2000 the minimal educational preparation for entry to practice of nursing be the successful completion of the MARN approved baccalaureate degree in nursing” [10]. In 1996 all the nursing diploma schools in the hospitals were closed and nursing education moved to the UM and Red River College. With nursing programs moved to postsecondary institutions, hospital libraries shifted focus from assisting nursing educational programs to supporting nurses on clinical rotations as well as clinical care.

Medical students in Winnipeg had traditionally performed their clinical placements in the two large teaching hospitals, the HSC and the SBGH. As medicine became more specialized, the large teaching hospitals became acute care centers. To provide students clinical access to a range of illnesses, a more distributed education model was adopted. More clinical placements were scheduled for community hospitals offering primary and secondary care. As a result, hospital libraries needed clinical materials for rotating medical students and other health care professionals.

In the 1990s, health care professionals found both new technologies and increasing quantities of published scientific literature daunting [11]. The need for training in information retrieval was pressing because many practitioners did not have the skills to find recent and accurate information. As McKibbon and Walker-Dilks [12] pointed out many were not even aware that their skills were deficient and little information literacy instruction was being delivered in hospital libraries.

#### c. *Political*

Political changes in the management of the healthcare system were also being implemented. In the early 1990s, regionalization of health services was sweeping the country. On May 14, 1992, the Manitoba Minister of Health issued the report “Quality Health for Manitobans – The Action Plan” [13]. Subsequently, Manitoba Health began decentralizing decision-making processes to Regional Health Authorities (RHAs). Thirteen RHAs were established including two in Winnipeg. In 2000 the two Winnipeg RHAs merged to form the Winnipeg Regional Health Authority (WRHA).

To achieve overall cost-effectiveness, the RHAs adopted a program management model. A basic principle of program management was to decant decisions to the least complex, most competent unit. Services not deemed primary functions (e.g. patient care) of the RHAs were evaluated and contracted to more appropriate agencies. Library services were considered non-primary functions. The newly formed RHAs had many challenges facing them and were aware that health professionals needed access to evidence-based information.

Similar technological, educational, and political challenges were being experienced across the country. A number of provincial initiatives emerged that focused on coordinating library services for health care providers working outside universities. Health Science Information Consortium of Toronto was “founded in 1990 out of a desire to strengthen the relationship between the University of Toronto Libraries and the libraries of health care institutions affiliated with the University's Faculty of Medicine” [14]. In the mid-1990s, the Health Knowledge Network (HKN) in Alberta initiated the provincial shared purchasing of electronic resources and the University of British Columbia’s Woodward Biomedical Library was managing teaching hospital libraries in the Vancouver area. The time was ripe for similar changes in Manitoba.

## Part 2: Revolution

The opening of the NJMSHL was a major catalyst for change in Winnipeg health library services. Its construction provided the initial infrastructure for service coordination. Changes to the administrative structure for the delivery of healthcare within Winnipeg also stimulated the expansion of library services across the city.

### Single Health Library for UM and HSC

A new medical library for Winnipeg had been planned since the 1970s and medical leaders repeatedly pushed for formal collaboration between area hospital libraries and the UM Medical Library. In the 1980s negotiations began between the Faculty of Medicine and HSC. HSC is the province’s largest teaching hospital and is located adjacent to the UM Health Sciences Campus (Bannatyne). A concerted effort was made to construct a new building which included a library to support both the Bannatyne campus and the HSC. HSC donated the land for the new building, the Brodie Centre.

In 1993, a formal agreement was signed between HSC and the UM integrating HSC Library Services into the future NJMHSL. The NJMHSL officially opened on June 5, 1996. As part of the merger, the HSC library technicians and librarian were given the option of transferring to the UM Libraries (UML). Funding was given for staff to manage three satellite collections – a Pediatrics Collection in the Children’s Hospital, a General Hospital Collection in the General Hospital, and a Psychiatric Collection in the Psych Health Centre. As a result of the successful merger, health professionals at the HSC had the best of all worlds - access to a world class university health science library and on-site clinical collections.

### St. Boniface Hospital Library Joins UM Libraries

In 1997, the SBGH, the city’s second largest teaching hospital, approached the UML requesting that its library become a NJMHSL satellite. It was well staffed with two professional librarians and 5.5 library assistants. Hospital administrators realized that they could not match the technology and resources of the UML. After a two-year negotiation, the SBGH Library became a unit of the NJMHSL. The technicians and librarians also transferred into the UML joining either the Association of Employees Supporting Education Services (AESES) or the University of Manitoba Faculty Association (UMFA) bargaining units.

### Victoria General Hospital Joins UM Libraries

In the mid-1990s, the Victoria General Hospital (VGH) was staffed with a library technician and ran a networked version of MEDLINE. Under former hospital leadership, the VGH Library was stripped of print materials because the perception was that most relevant medical information was available on the internet. The VGH Library limped along for a number of years until a professional librarian was hired in the library technician’s position. Although the hospital did not increase the materials budget, the librarian offered a higher level of service. When the librarian left in 1998, he wrote a report with a number of recommendations including that the VGH commit to hiring a professional librarian [15]. In August 1998, a new VGH CEO approached the UML and requested an assessment of the library. The assessment [16] led to an invitation from VGH for the UML to manage library services. In April 1999, negotiations were concluded, and an agreement was signed which included funding for a .5 librarian, a .5 technician, and library materials. Both positions were brought in as UML employees, and it was agreed that additional VGH funding would be provided in due course to increase staffing and library resources.

### The Subsidiary Affiliation Agreement – Libraries

The success of these agreements inspired other Winnipeg hospitals to advocate for similar arrangements. Senior management within the Winnipeg region was aware of the library service agreements negotiated between the UML and the three hospitals. Separate negotiations were considered time consuming and not representative of the program management approach. In 2000, the newly formed WRHA approached the University and requested an agreement covering library services for all Winnipeg hospitals.

The *Subsidiary Affiliation Agreement – Libraries* was written and signed in 2000 by the University and WRHA [17]. The Agreement addressed the philosophy of library services (i.e. a program management model) and stipulated service assessments be conducted for each Winnipeg hospital and health care centre. WRHA senior management made library services for the three remaining hospitals (Concordia, Seven Oaks, and Grace) a priority. Each library was assessed with a view to transferring management to the NJMHSL but hospitals could opt out. As with the earlier assessments, reports were written considering differences in funding, staffing, collections, and technology at each facility [18,19,20]. Completed assessments were reviewed by the University, the WRHA and the CEOs of each hospital. The service assessments were the basis of a final agreement. One-time baseline funds were transferred from the Minister of Health to the Minister of Education and then to the University and the UML. This ensured that the hospital libraries and services were funded through the UM. The implementation of this agreement changed the fundamental structure of both the hospital libraries and the NJMHSL. A joint UM/WRHA Library Program Liaison Committee (LPLC) was established to provide oversight and to ensure that individual and joint obligations were met. The LPLC was structured to function at high level within both the UM and the WRHA. It was co-chaired by the head of the Health Sciences Libraries, who reported to the University Librarian, and the Executive Director of Research and Applied Learning, a physician, who reported to the CEO of the WRHA. Membership in this committee included the UM President (or their representative), the University Librarian, and representatives from the WRHA clinical departments including medicine, nursing, and allied health. The committee met regularly, and reports were sent to them throughout the year. The committee was also asked to make presentations to the WRHA Leadership Council. The LPLC worked well at keeping everyone appraised of developments and changes. The WRHA was very supportive as evidenced by funding new stages of the program.

### Grace, Seven Oaks, and Concordia Hospitals Join UM Libraries

In 2002, the Grace, Seven Oaks, and Concordia Hospital libraries became satellites of NJMHSL. Each satellite had core budget requirements ensuring they would have annual funding for:

- One FTE librarian and FTE library technician
- A monograph budget of $10,000 at each location
- A serials budget (variable at each location) transferred to a central fund for journal acquisition
- A technology budget of $10,000 at each location
- A supplies budget of $4000 at each location
- An administrative budget for the UML
- One full-time UML IT staff member to support all locations

The WRHA insisted that no existing library staff lose their positions– and no one did. Meetings were held with human resources departments in each hospital, hospital unions representing library staff, and the UM AESES bargaining unit. Although pensions could not be transferred to the University’s fund, seniority was maintained. All five hospital libraries and the NJMHSL became known as the UM Health Sciences Libraries (HSL).

### Library Services for WRHA Community Services and Long-Term Care

Senior management in the WRHA recognized that staff working outside the hospitals also required library services. The WRHA subsequently asked the UM to consider consolidating the three health centre libraries focused on rehabilitation and long-term care. As a result, the Deer Lodge Health Centre, Misericordia Health Centre and Riverview Health Centre libraries became part of the HSL. A separate agreement was not required for this development, but a report was requested. The report, *Library Services for Long Term Care in the Regional Health Authority*, included details on staff, funding, resources and services [21]. The preamble of the initial *Subsidiary Affiliation Agreement – Libraries* also included a Schedule C listing the names of thirty-five personal care homes, fourteen community health agencies, and fourteen mental health agencies that should be considered for future library services. Providing service to the personal care homes was included in the *Library Services for Long Term Care* report and funding was provided by the WRHA [21].

No additional negotiations took place for the community health agencies and mental health agencies listed in Schedule C. In a *Letter of Agreement - Library Services*, written by the CEO of the WRHA in 2008, the issue of service to the community and mental health agencies was addressed [22]. This letter included a paragraph which stated that “the WRHA and the University further agree to work collaboratively to pursue a provincial license relating to access to electronic health databases, articles and communication networks”. The HSL had the capacity to provide services to these organizations because a low number of service requests were anticipated. Hospital librarians became responsible for services to specific community-based health facilities. Services to these facilities included access to the UML collection, literature search requests, document delivery, and training. An *Amending Agreement* was drafted by WRHA which would have seen the term “WRHA Hospital” replaced by the term “WRHA Organization” and would have included the community agencies and mental health agencies. The *Amending Agreement* was never signed by the University Librarian, a fact of critical importance in later developments.

### Health Sciences Libraries 2004-2014

By 2006, all the WRHA hospitals and health care centres had been integrated into the UML and the HSL was structured as follows:

- The NJMHSL was part of the UML structure
- One main academic health sciences library – the NJMHSL - that also served the largest teaching hospital, HSC
- Five hospital libraries – Seven Oaks, Victoria, St. Boniface, Grace, and Concordia
- Three health centre libraries – Deer Lodge, Riverview, and Misericordia
- Outreach services to:
  - WRHA Corporate Office, WRHA community area offices, and ACCESS Centres (centres providing primary care and family services)
  - Community health and mental health agencies (twenty-eight in total)
  - Thirty-five personal care homes

**Figure 1:**
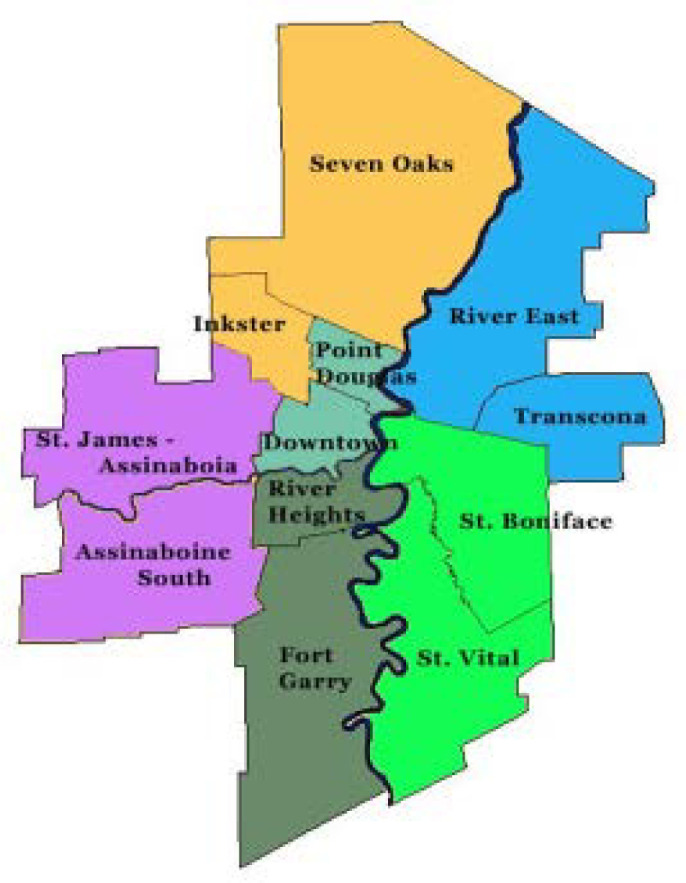
WRHA Community Areas & Associated UM Health Libraries

The staff complement included 10.5 academic librarians and 9.5 technicians. To ensure that librarian workload was distributed evenly and that all areas of the city were effectively serviced, eight access points were recognized as outlined in Figure 1.

In addition to the HSL service network, a few other developments rounded out the provision of library services to the WRHA. The WRHA Corporate Office was not originally considered in the *Subsidiary Affiliation Agreement - Libraries* but there were staff located in these offices conducting research and writing health policy often in collaboration with UM faculty. A 2005 study revealed that more librarian support was needed and the HSL reconfigured existing staffing so that a .5 librarian position provided support to the Corporate Office [23]. In addition, a subsequent proposal to the CEO of the WRHA requested funding to cover some staffing shortfalls. The Misericordia Health Centre librarian position was extended to a full-time appointment with the view that this position could also provide services to community health agencies in Point Douglas, Inkster and the Downtown area. An additional librarian position was added to the SBGH Library to provide assistance to the community agencies and liaise with the UM Family Medicine program at SBGH.

**VGH Library** – the hospital and WRHA staff in Community Areas of Ft. Garry and River Heights

**SBGH Library** – the hospital, UM Family Medicine program at SBGH and WRHA staff in Community Areas of St. Boniface and St. Vital

**Concordia Hospital Library** – the hospital and WRHA staff in Community Area of Transcona

**Seven Oaks General Hospital Library** – the hospital, UM Family Medicine program at Seven Oaks, and WRHA staff in Community Areas of Seven Oaks and River East

**Grace Hospital Library** – the hospital and WRHA staff in Community Areas of St. James Assiniboia and Assiniboine South

**J.W. Crane Memorial Library (Deer Lodge Centre)** – the centre, the Riverview Health Centre and 35 Winnipeg personal care homes

**Misericordia Health Centre Library** – the centre, the Health Links program (a nurse led telephone triaging service), and Community Areas of Downtown, Point Douglas, and Inkster

**Neil John Maclean Health Sciences Library** – UM Bannatyne Campus, HSC and WRHA Corporate Office

### Success with the HSL Service Model

Between 1995 and 2015, many consortia were established, and agreements signed between hospitals and university health libraries across the country [1, 2]. The UM HSL service mModel was unique in Canada because all, both teaching and community, hospital libraries in the city were integrated and became satellite libraries of the UML. In retrospect, a number of critical factors facilitated this development:

1. Support for the initiative came from the highest levels of the University and WRHA;
2. *A Master Affiliation Agreement* had already been negotiated between the University and the WRHA serving as an umbrella under which subsequent agreements could be established;
3. Knowledge-based information was largely print based, and agreements were simpler to negotiate because at the time publishers’ electronic licenses were less of a factor;
4. Early successes with the HSC and the SBGH resulted in champions and supporters who encouraged the WRHA to look at extending the model throughout Winnipeg;
5. The model supported the WRHA’s strategic goal of developing a “learning organization with a strong safety culture and timely access to accurate and relevant information to support decision makers” [24];
6. The WRHA was committed to implementing a program management approach for the delivery of services and the *Agreement* was an example of one that worked.
7. UML and WRHA administrators developed relationships over a long period of time resulting in consistent planning and development. There was buy-in from hospital CEOs and no institution was forced to participate;
8. A reasonable amount of funding was requested with recommended operational and staffing standards. The Minister of Health approved and cooperated with the Minister of Education to transfer funds to the University.

The development of the HSL Service Model was not rushed. The model included taking the time needed to consult staff at all levels of the various organizations; gathering information; and making strategic plans. Frequent meetings were scheduled to reassure affected staff that their concerns and contributions were being taken seriously. Time was spent with the existing hospital library staff to discuss the gains and benefits of collaboration. Staff were asked to concentrate not on what they had lost but to recognize the tension between centralized and decentralized services. Everyone cooperated in crafting a set of guiding principles to establish an overall plan of service delivery, resource acquisition, and staffing for the region. In the smaller libraries, service was paramount, but the opportunity to become part of a larger health libraries system offered increased access to of resources and technology for clients. The model incorporated the best of both worlds. All of these factors were enhanced by good timing, hard work, persistence and luck.

### Assessments of the HSL Service Model 2006, 2011, and 2013

Three large surveys were undertaken over the years, to ensure that the HSL was providing the services that clients wanted and to assess service quality. An initial WRHA Library Satisfaction Survey was conducted in 2006. The health administrators who participated indicated satisfaction with the HSL services and resources. In 2011, the HSL participated in the *Value of Library and Information Services in Patient Care Study* funded by the National Library of Medicine [25]. The HSL was one of only four Canadian sites in a group of 56. This survey was a replication of the landmark Rochester study [26] and reconfirmed that “information obtained from a library had an impact on patient care” [25]. In 2013, the initial 2006 survey was replicated but distributed more widely to health care professionals across the region [27]. About 1,000 healthcare providers participated in the survey with 57% (570) having used HSL services. The results demonstrated that administrators, managers, and healthcare professionals rated online databases and online journals more highly and used them more frequently than point-of-care tools. The most highly used and highly ranked library services were librarian-mediated literature searches, document delivery, and the ability to link to full-text journal articles. These results were echoed in the many qualitative comments from respondents stating how the services saved time and made them more efficient. Assessments revealed that the model was working to support health providers throughout the region.

## Part 3: Devolution

The organizational structure set in place allowed for the effective and efficient functioning of the HSL. Despite this, the many successes of the HSL Services Model were offset by significant challenges that led to radical changes. Factors that contributed to the changes were recognized early in the development of the model. These included: electronic access to resources; creation of a unique category for WRHA employees in the integrated library system (ILS); license requirements limiting access for WRHA employees; issues with baselined funds; and librarian academic appointments. Unanticipated factors included political change; shifts in the philosophy of academic library service to the community; and limitations in lifespans of agreements between organizations.

### Electronic Access to Resources

The *Agreement* between the UM and WRHA was written when print collections dominated and services in the hospital libraries were focused on reference assistance; mediated literature searches; faxing of journal articles; mail delivery of books and audiovisual materials; and basic instruction on MEDLINE, CINAHL, and the internet [17]. Few had heard of Google.

Although this may seem naïve today, the original *Agreement* granted WRHA staff online access to UML electronic resources **only** if they visited a hospital library. This agreement was adequate at the time because there were few e-journals, no e-books, and PubMed had become freely available online. Most publisher licences allowed for “drop-in access” to UML online resources. Electronic journals were just starting to emerge and large-scale licensing of electronic products was in its infancy. WHRA nurses, allied health professionals, and other hospital staff without academic appointments were considered drop-in users. Physicians with admitting privileges in Manitoba hospitals had academic appointments with the University giving them access to all UML resources.

Licensing of online resources became more of an issue as resources rapidly migrated online. WRHA staff were no longer considered “drop in” users and publishers began demanding expanded licenses from the UML. The *Agreement* was unclear and the wording that appeared under article 3.1.c, *Obligations of the University*, stated [17]:

…grant to the WRHA Medical Staff and to staff of the WRHA working within the head office of the WRHA, the WRHA Head Office Staff and to all staff of WRHA Participating Hospitals access to all library facilities and related services established, by the University, from time to time, in the WRHA Hospital Libraries, on the same terms and conditions as said library facilities and related services are made available to the University’s staff and students.

More importantly item 3.1.d.iii stated “Access to the online catalogue and the full line of electronic services via the UML computer networks” [17]. Under these terms, the UML was obligated to provide access to its online resources to WRHA “head office” and all Winnipeg hospital staff.

### Creating a Unique Category in the ILS

Issues with the *Agreement* and electronic access were further compounded by the category assigned to WRHA staff in the ILS. The UM ILS was available in all the Winnipeg hospital libraries. WRHA staff were given special borrower cards that permitted searching of the ILS; borrowing of UML print materials; and logging into hospital library computers to access UML licensed databases and the internet. Early in the evolution of the HSL Library Services Model the Head of the UM HSL Library Services Program recommended the addition of a unique category for WRHA staff so they could be easily identified and access to electronic resources restricted, as needed. The recommendation was not implemented by UML administration.

As a result, WRHA staff were registered in the UM Faculty/Staff/Student category and automatically had full access to all UML electronic resources. The WRHA Senior Executive and staff were repeatedly reminded and they acknowledged that offsite access to UML electronic resources for WRHA staff was not part of the *Agreement* and that access could be lost at any time. In recognition of these licensing difficulties, the UML began negotiating access for WRHA staff. In some cases, vendors added the WRHA staff with little or no additional cost. In other cases, the costs were prohibitive since the WRHA had over 28,000 employees.

In 2012, the WRHA was informed that only staff working in the hospitals and the Corporate Office building would be covered in licensing agreements for electronic resources. This decision left many WRHA staff without access to resources on which they had become reliant. This have and have-not scenario caused resentment amongst staff. There were many complaints to the WRHA leadership. Eventually a new University Librarian (UL) would revisit the issue as the UM faced a period of fiscal restraint.

### Academic Appointments

Librarian staffing presented an additional problem. At the UM, librarians hold academic appointments. When librarians were hired they were eligible for continuing appointments with a variety of benefits including research leaves. The *University of Manitoba Faculty Association Collective Agreement* (UMFA Article 21) states that librarians are able to take a full year of research leave for every six years worked [28]. The hospital libraries employed 10.5 UML librarians. This meant that as many as two librarians per year could be on research leave. In hindsight, provisions should have been made in the *Agreement* for invoicing the WRHA for the cost of leave replacement librarians. Initially the UML covered the costs but as time went on budgets were increasingly strained and new UML leadership could not justify them. After a review by UM Human Resources in 2013, it was determined that new hospital librarian hires would be offered “contingent appointments” and ineligible for research leaves.

### Funding of the HSL Library Services Model

Similar to the issues with online access, the entire funding model gradually became problematic. The original *Agreement* obligated the WRHA to transfer one-time baseline funds to the University for the provision of library services. The flow of the one-time funds was from the Minister of Health to the Minister of Education and then to the University budget. The understanding of baseline funding hinged on the definition in university documentation that indicated this meant permanent ongoing budget amounts for the UML [29]. It was assumed that once the WRHA money for library services became baselined to the University that the hospital libraries became University libraries and, going forward would receive the same increases as all other departments.

And, in fact, that is the way the UML managed the hospital libraries until 2008. However, there was no stipulation in the *Agreement* that once the money was baselined to the University that they were obligated to provide yearly increases. University funding comes from many sources (Manitoba Education and Training (MET), tuition and related fees, ancillary fees, sales of goods and services, investments, and other grants from the Province of Manitoba and the Government of Canada). Of all these funding sources, only the allocation from MET has a negotiated annual increase. Since the operating grant from MET represented approximately fifty-nine percent of UM total operating revenues, the baseline funding from the WRHA to the UM eroded while salaries and acquisition budgets increased.

In retrospect, the baselined funds issue should have been a red flag to everyone negotiating the *Agreement*, but most specifically to University administrators and legal advisors. Years later, senior administrators in the WRHA stated that the *Agreement* could have had provisions built into it which would have seen the WRHA provide yearly increases for cost-of-living adjustments, salary increases, and journal cost increases. It would have been easier for the UML and WRHA to obtain funding from Manitoba Health during the years that the New Democratic Party (NDP) was in power (1999 to 2016). However, the University did not request these increases and they became an issue when UML leadership changed, and finances became increasingly constrained under a Progressive Conservative (PC) government.

### Political and Financial Change

In 2017, several changes took place that necessitated a change to the *Agreement*. The PCs were elected in 2016 and introduced a deficit reducing budget. The WRHA was told to cut eighty-three million dollars from its operating funds. At the same time, the WRHA staff who did not have access to UML electronic resources were putting pressure on the WRHA administration to negotiate electronic access to health-related resources. This would necessitate a change in the license agreements and additional costs. Given the cuts demanded by the government, the WRHA was not in a position to increase the budget for HSL Library Services.

The UM was also experiencing budget cuts and, in 2016, the institution embarked on a redesigned budget model. The new budget model changed the way funds were transferred to faculties encouraging them to become more creative in generating income. UM faculties generated income by establishing articulation agreements with universities in other countries to attract international students and also by establishing new graduate programs with higher tuition fees. The UML administration had fewer options for generating income but viewed contracts and affiliation agreements for library services as possibilities.

Because of the PC government’s austerity platform; the UM budget redesign; and the UML’s new interest in generating income, the WRHA and the UM revisited the *Agreement*.

### Philosophy of Service

University Librarians (ULs) have different philosophies of service. Their views, experiences, and financial environments impact the extent to which they are willing to implement external agreements. Three ULs were part of the lifespan of the HSL Service Model. The UL in 1990 – 2009 had an inclusive view of library services and believed the UML was a provincial leader in information delivery. They also understood that the *Agreement* benefited UM staff and students working and learning in Winnipeg healthcare facilities.

The UL in 2009 – 2014 was faced with the electronic access issues and a different financial reality. Their risk management response was to limit access to electronic resources for the WRHA staff. Up until 2015, the health sciences librarians in both the hospitals and the University worked collaboratively to provide services to UM faculty and students and to WRHA staff. This was a fluid and reciprocal environment in which the hospital librarians participated in delivering training sessions to university faculty and students and the university librarians assisted with literature searches for WRHA staff.

The UL in 2014 – 2018 implemented a strict demarcation between the support provided to the “academy” and the external support provided to WRHA staff. They also advocated for a shift in services from mediated to self-service. Both these changes radically altered the HSL Services Model [30].

In addition to the above, other issues contributed to the changes. The UL who served from 1990-2009 was fully committed to maintaining the structure as it had been developed. The two subsequent ULs did not fully support the purpose and shared vision of the HSL Library Service Model [31], as the people who developed it. There may also have been the perception of an imbalance of power wherein the WRHA received the greater benefits while the UML incurred the greater risk [32].

### Agreement Lifespans

Do agreements have a lifespan? [33,34] Probably. Some agreements come to a close; others evolve, while others are resurrected after a number of years under different but similar mandates. For example, Saskatchewan Health Information Resources Program (SHIRP) changed from a provincial partnership to a University of Saskatchewan program [35]. In Manitoba, a number of library agreements came to a close over the years. For example, the College of Physicians and Surgeons *Extension Service* agreement for rural doctors negotiated with the former UM Medical Library lasted seventeen years. Similarly, the *Manitoba Health Information Network (MHINET)* agreement with Manitoba Association of Registered Nurse lasted thirteen years. In total, all of the agreements with the hospitals and WRHA lasted about twenty years. Given the complexities of the environment and the various pressures under which it operated, the HSL Library Services Model made a significant contribution to healthcare provision in Manitoba [27].

### A New Model – The WRHA Virtual Library

As these factors converged and the HSL Library Services Model devolved, a new service model was needed. On January 1, 2018, the UL officially announced that the eight WRHA hospital libraries would be closed, and the service transitioned to a new service, the WRHA Virtual Library. The savings from hospital library closures and staff reductions were used to purchase licenses for electronic resources specifically for the WRHA.

The staff complement for the new service model included four librarians and four library technicians who continue to be located at the NJMHSL [30]. No librarians or technicians lost their jobs during this transition as they were reallocated to vacant positions within the UML. One librarian, the WRHA Electronic Services Librarian, was relocated to the Fort Garry Campus, with three librarians and four technicians providing the services formerly delivered by twenty people. Unlike the old model, the WRHA Virtual Library Services was not managed by the Head of the NJMHSL but instead an Associate University Librarian (AUL) overseeing all UML contracted services.

The evolving library service continues to have successes and challenges [30]. Although the WRHA sought electronic access for all 28,000 employees, this is not yet a reality. The original Subsidiary Affiliation Agreement – Libraries stipulated WRHA staff would be given access to all UML resources, whereas the new WRHA Virtual Library portal provides access to freely available online resources and a select suite of subscription resources.

## Discussion

Access to evidence-based health information and library services for healthcare providers has long been a challenging issue in Canada. Major barriers identified by the Canadian Health Libraries Association (CHLA/ABSC) to establishing a national solution included: the fact that healthcare is a provincial responsibility; the costs of establishing a new body to coordinate a virtual library; and the costs of acquiring evidence-based resources [37]. For over a decade, the CHLA/ABSC attempted to address the issue nationally. From 2000 to 2008, stakeholders across the country were consulted culminating in the funding and creation of a Canadian Virtual Health Library in 2010, a project that lasted approximately two years [38]. Meanwhile across the country, various provincial initiatives developed, some still extant and developing, some devolved. Winnipeg’s HSL Library Services Model and new WRHA Virtual Health Library offers lessons for existing and future services.

### a. *Agreements*

While cooperation between universities and government agencies seems like a “win- win” proposition, this paper reveals their fragility and complexity. The fact is that agreements are “living” documents and need monitoring and revision to remain relevant. High level communication between the UL and the senior administration within the WRHA was lacking in the latter years of the HSL Library Services Program. Changes in leadership on both sides contributed to a loss of continuity in understanding the history of the relationship and a commitment to the *Agreement*. Additional mechanisms, such as a requirement for a biennial review of the *Agreement*, could have been written into the *Agreement* to ensure long-term sustainability and to allow library services to evolve gradually.

### b. *Long term Fiscal Planning*

While funding had been addressed in the early stages of the development of the *Agreement*, unforeseen internal and external financial challenges eventually had an impact on the service. A consistent reexamination of the *Agreement* including regular reviews by the financial departments of each organization would have provided snapshots that anticipated budgetary problems.

### c. *Evolving Nature of Evidence-Based Healthcare Information*

The HSL Library Services Model as originally conceived, involved librarians providing mediated searches and reference consultation service. The services were heavily used as evidenced by a 2015 analysis of the searches performed by the HSL librarians. From 2004 to 2010, approximately 19,000 searches were conducted with each search taking an average of 85 minutes [39]. Although many WRHA staff also had online access to a variety of evidence-based resources during this time, they preferred mediated services because it saved them time and they received a quality product. As evidence-based medicine products incorporate more algorithms and artificial intelligence, the need for mediated searching for day-to-day information seeking becomes less necessary [ 40,41,42]. The need for research support for systematic reviews or broader investigations of changes to support patient care within the healthcare system still remains. This support continues to be provided by librarians in the WRHA Virtual Library [30].

### d. *Recentralization of Provincial Healthcare Services*

Experiments with decentralization through regionalization continue, with some provinces reducing the number of regions, others recentralizing delivery. For example, Alberta has become one large health region and has developed library services, the Knowledge Resource Service, for the healthcare providers across the province [43]. Manitoba is also quickly moving towards a single regional health authority, Shared Services. These organizational shifts require continuous reconfiguration of library service delivery and point to a different model for the provision of information services for healthcare providers. Integrating evidence-based health information into provincial electronic health records is the future [41]. As things currently stand, this type of access cannot be delivered by a university academic library system. It might be reasonable to integrate knowledge sources into electronic health records through provincial regional models or perhaps a consortial membership model. However, electronic record linkage to knowledge sources is still under development, with evidence of success still needed.

### e. *Ongoing Need for National Access to Evidence-Based Healthcare Information*

The provincial challenges underscore that national access to evidence-based healthcare information continues to be fragmented. Within each province, differing sets of products and services are available to healthcare providers and varies by profession. National associations like the Canadian Medical Association and the Canadian Nurses Association attempt to compensate for this differentiation by offering their members access to various evidence-based resources. Some health care providers (e.g. Physician Assistants) belong to associations who do not have the financial resources to offer their members such benefits. Studies have shown that better and more cost-effective decisions are made when healthcare providers have access to high quality evidence at the point of care [25]. A recent drive for a national Pharmacare Plan underscores yet again the need for some kind of national access to a suite of relevant evidence-based healthcare tools including drug/pharmaceutical databases.

## Conclusion

From the early evolution of health library services in Manitoba, a confluence of factors resulted in the HSL, a revolutionary library service model that served Winnipeg health professionals for nearly 20 years. Part of this success was the inextricable link between the UM and WRHA clinical programs with one medical school adjacent to one large provincial hospital located in one major city. The HSL library services to the WRHA have devolved since 2018 due to recognized and unanticipated factors. However, a new WRHA Virtual Library model has emerged. Data will be gathered and analyzed as the service develops. Time will tell if it is meeting the needs of Winnipeg healthcare providers but the lifespan of agreements, long term financial planning, the evolving nature of evidence-based health information, the recentralization of provincial healthcare services, and the ongoing need for a pan-Canadian access to evidence-based health information may be factors in its future.
